# Fluxametamide (Pesticides)

**DOI:** 10.14252/foodsafetyfscj.D-20-00006

**Published:** 2020-03-27

**Authors:** Food Safety Commission of Japan

## Abstract

FSCJ conducted a risk assessment of fluxametamide (CAS No. 928783-29-3), an isoxazoline insecticide, based on results from various studies. The data used in the assessment include the fate in animals, fate in plants, residues in crops, subacute toxicity, subacute neurotoxicity, chronic toxicity, combined chronic toxicity/carcinogenicity, carcinogenicity, two-generation reproductive toxicity, developmental toxicity, and genotoxicity. Alveolar macrophage accumulation, vacuolated epithelial cells in the small intestine, and hepatocellular vacuolation are observed in various toxicity studies. Increased incidences of thyroid follicular cell adenoma in male rats and of hepatocellular adenoma in male mice were observed in carcinogenicity studies. However, a genotoxic mechanism was unlikely to be involved in the tumor increases. FSCJ specified an acceptable daily intake (ADI) of 0.0085 mg/kg bw per day, applying a safety factor of 100 to the NOAEL, 0.85 mg/kg bw per day, that was derived from the two-year combined chronic toxicity/carcinogenicity study in rats.

## Conclusion in brief

FSCJ conducted a risk assessment of fluxametamide (CAS No. 928783-29-3), an isoxazoline insecticide, based on results from various studies.

The data used in the assessment include the fate in animals (rats), fate in plants (strawberries, egg plants and others), residues in crops, subacute toxicity (rats and dogs), subacute neurotoxicity (rats), chronic toxicity (dogs), combined chronic toxicity/carcinogenicity (rats), carcinogenicity (mice), two-generation reproductive toxicity (rats), developmental toxicity (rats and rabbits), and genotoxicity.

Alveolar macrophage accumulation, vacuolated epithelial cells in the small intestine, and hepatocellular vacuolation are observed in various toxicity studies of fluxametamide. Fluxametamide showed neither neurotoxicity, reproductive toxicity, teratogenicity nor genotoxicity.

Increased incidences of thyroid follicular cell adenoma in male rats and of hepatocellular adenoma in male mice were observed in carcinogenicity studies. However, a genotoxic mechanism was unlikely to be involved in the tumor increases. It was thus reasonably considered to establish a threshold dose in the assessment.

Based on various studies, fluxametamide (parent compound only) was identified as the relevant substance for the residue definition for dietary risk assessment in agricultural products.

The lowest no-observed-adverse-effect level (NOAEL) obtained in all studies was 0.85 mg/kg bw per day in the two-year combined chronic toxicity/carcinogenicity study in rats. FSCJ specified an acceptable daily intake (ADI) of 0.0085 mg/kg bw per day, applying a safety factor of 100 to the NOAEL.

FSCJ considered it unnecessary to specify an acute reference dose (ARfD), since fluxametamide is unlikely to exert toxic effects after a single oral dose administration. (table 1)

**Table 1.** *Levels relevant to toxicological evaluation of fluxametamide*

Species	Study	Dose (mg/kg bw/day)	NOAEL (mg/kg bw/day)	LOAEL (mg/kg bw/day)	NOAEL^1)^
Rat	90-day subacute toxicity study	0, 200, 2 000, 20 000 ppm	M: 14 F: 17	M: 140 F: 174	FM: Vacuolated intestinal epithelial cells, alveolar macrophage accumulation and others
		M: 0, 14, 140, 1 430 F: 0, 17, 174, 1 670			
	90-day subacute neurotoxicity study	0, 160, 1 600, 16 000 ppm	M: 102 F: 121	M: 1 030 F: 1 190	FM: Vacuolated intestinal epithelial cells (No subacute neurotoxicity)
		M: 0, 9.96, 102, 1 030 F: 0, 12.2, 121, 1 190			
	Two-year combined chronic toxicity/carcinogenicity study	0, 20, 200, 2 000, 20 000 ppm	M: 0.85 F: 1.2	M: 8.6 F: 12.1	FM: Centrilobular hepatocellular vacuolation and others (FM: Thyroid follicular cell adenomas)
		M: 0, 0.85, 8.6, 89, 899 F: 0, 1.2, 12.1, 120, 1 250			
	Two-generation reproductive toxicity study	0, 10, 20, 60, 200 ppm	Parent PM: 4.7 PF: 18.2 F_1_M: 5.5 F_1_F: 20.1 Offsprings PM: 1.6 PF: 5.5 F_1_M: 1.9 F_1_F: 6.2	Parent: PM: 16.2 PF: - F_1_M: 19.2 F_1_F: - Offsprings PM: 4.7 PF: 18.2 F_1_M: 5.5 F_1_F: 20.1	Parent M: Increase in the percentage of morphologically abnormal sperm and others F: No toxicological effect Offsprings M: Delayed preputial separation F: Abdominal distension and others (No effect on reproduction)
		PM: 0, 0.82, 1.6, 4.7, 16.2 PF: 0, 0.90, 1.8, 5.5, 18.2 F_1_M: 0, 0.97, 1.9, 5.5, 19.2 F_1_F: 0, 1.11, 2.1, 6.2, 20.1			
	Developmental toxicity study	0, 100, 300, 1 000	Maternal: 1 000 Embryo/fetus: 100	Maternal: - Embryo/fetus: 300	Maternal: No toxicological effect Embryo/fetus: Supernumerary ribs and others (Not teratogenic)
Mouse	18-month carcinogenicity study	0, 10, 100, 1 000, 8 000 ppm	M: 0.99 F: 11.1	M: 10.1 F: 114	FM: Liver effects including increased absolute/relative liver weight (M: Hepatocellular adenomas)
		M: 0, 0.99, 10.1, 104, 877 F: 0, 1.10, 11.1, 114, 951			
Rabbit	Developmental toxicity study	0, 100, 300, 1 000	Maternal: 300 Embryo/fetus: 300	Maternal: 1000 Embryo/fetus: 1000	Maternal: Suppressed body weight and others Embryo/fetus: Abnormal lobation of the lungs and others (Not teratogenic)
Dog	90-day subacute toxicity study	0, 100, 300, 1 000	M: 1000 F: 1000	M: - F: -	FM: No toxicological effect
	One-year chronic toxicity study	0, 10, 100, 1 000	M: 100 F: 100	M: 1 000 F: 1 000	FM: Decrease 33 in T.Chol and others
ADI			NOAEL: 0.85 SF: 100 ADI: 0.0085		
The critical study for setting the ADI			Two-year combined chronic toxicity/carcinogenicity study in rats		

ADI, Acceptable daily intake; SF, Safety factor; NOAEL, No-observed-adverse-effect level

-, NOAEL could not be specified

^1^, The adverse effect observed at NOAEL

